# Berberine enhances inhibition of glioma tumor cell migration and invasiveness mediated by arsenic trioxide

**DOI:** 10.1186/1471-2407-8-58

**Published:** 2008-02-25

**Authors:** Tseng-Hsi Lin, Hsing-Chun Kuo, Fen-Pi Chou, Fung-Jou Lu

**Affiliations:** 1 Institute of Biochemistry and Biotechnology, Chung Shan Medical University, Taichung 402, Taiwan; 2 School of Applied Chemistry, Chung Shan Medical University, Taichung, Taiwan; 3 Division of Hematology, Department of Internal Medicine, Taichung Veterans General Hospital, Taichung, Taiwan; 4 Department of Medical Research, Chang Gung Memorial Hospital, Kaohsiung Medical Center, Kaohsiung 833, Taiwan

## Abstract

**Background:**

Arsenic trioxide (As_2_O_3_) exhibits promising anticarcinogenic activity in acute promyelocytic leukemic patients and induces apoptosis in various tumor cells *in vitro*. Here, we investigated the effect of the natural alkaloid berberine on As_2_O_3_-mediated inhibition of cancer cell migration using rat and human glioma cell lines.

**Methods:**

The 3-(4,5-dimethylthiazol-2-yl)-2,5-diphenyl tetrazolium bromide (MTT) assay was used to determine the viability of rat C6 and human U-87 glioma cells after treatment with As_2_O_3 _or berberine, and after co-treatment with As_2_O_3 _and berberine. The wound scratch and Boyden chamber assays were applied to determine the effect of As_2_O_3 _and berberine on the migration capacity and invasiveness of glioma cancer cells. Zymography and Western blot analyses provided information on the effect of As_2_O_3 _and berberine on the intracellular translocation and activation of protein kinase C (PKC), and some PKC-related downstream factors. Most assays were performed three times, independently, and data were analyzed using ANOVA.

**Results:**

The cell viability studies demonstrated that berberine enhances As_2_O_3_-mediated inhibition of glioma cell growth after 24 h incubation. Untreated control cells formed a confluent layer, the formation of which was inhibited upon incubation with 5 μM As_2_O_3_. The latter effect was even more pronounced in the presence of 10 μM berberine. The As_2_O_3_-mediated reduction in motility and invasion of glioma cells was enhanced upon co-treatment with berberine. Furthermore, it has been reported that PKC isoforms influence the morphology of the actin cytoskeleton, as well as the activation of metalloproteases MT1-MMP and MMP-2, reported to be involved in cancer cell migration. Treatment of glioma cells with As_2_O_3 _and berberine significantly decreased the activation of PKC α and ε and led to actin cytoskeleton rearrangements. The levels of two downstream transcription factors, myc and jun, and MT1-MMP and MMP-2 were also significantly reduced.

**Conclusion:**

Upon co-treatment of glioma cells with As_2_O_3 _and berberine, cancer cell metastasis can be significantly inhibited, most likely by blocking the PKC-mediated signaling pathway involved in cancer cell migration. This study is potentially interesting for the development of novel chemotherapeutic approaches in the treatment of malignant gliomas and cancer development in general.

## Background

Arsenic trioxide (As_2_O_3_) can effectively induce apoptosis in acute promyelocytic leukemia (APL) cells *in vitro *and *in vivo *[1-4] and was approved by the United States Food and Drug Administration in 2000 for the treatment of patients with relapsed/refractory APL. Although As_2_O_3 _has been evaluated in clinical studies for the treatment of acute myelogenous leukemia, myelodysplastic syndrome, and multiple myeloma [5], the diverse sensitivities of different types of tumor cells to this drug limits its clinical application in a wider spectrum of hematological and, especially, solid malignancies [6-12]. The antiproliferation mechanisms of As_2_O_3 _in solid tumors are not well known, and studies on the anti-invasive effects are rare [13]. Most recently, As_2_O_3 _was reported to induce apoptosis in a human neuroblastoma cell line via the upregulation of caspase 3 [14]. Paradoxically, arsenic compounds are well-known human carcinogens that may cause, at relatively high concentrations and/or exposures times [15], tumors in a variety of human tissues including skin, liver, and kidneys [10].

The poor prognosis of human malignant gliomas is due to their invasion and recurrence. The invasion of glioma into normal brain tissue is a major challenge to clinical intervention because these tumors often highly infiltrate the surrounding brain tissues. An important characteristic of high-grade central nervous system tumors is the presence of massively upregulated protein kinase C (PKC) when compared to normal glia [16,17]. PKC represents a family of lipid-dependent serine/threonine kinases that consist of at least 12 mammalian isoforms divided into three subfamilies including conventional or classic PKCs (cPKC), non-classic or novel PKCs (nPKC), and atypical PKCs [18]. The activation of most PKC isoforms depends on the translocation from the cytosol to subcellular compartments such as the cell membrane [19]. The enhanced PKC levels in glioma cells have been suggested to be critical to the hyper-proliferative state and the resistance to apoptosis as well as glioma invasion [20,21]. Indeed, treatment of the human glioblastoma cell line T98G with hypericin results in a significant inhibition of the cell invasion, an effect that is also obtained using specific PKC inhibitors [22], and a high level of PKCα expression in a human colon-adenocarcinoma cell line has been correlated with high migratory activity of colon carcinoma cells [23]. Consequently, specific PKC inhibition is thought to control tumor growth and development [18,24]. PKCα/β inhibitor Go6976 blocks the invasion of urinary bladder carcinoma cells [25] and PKC antisense oligonucleotide LY900003 is in clinical development as a drug against breast cancer to be used in concert with, for instance, chemotherapy [26].

Tumor invasion including that of high-grade malignant gliomas is, for a significant part, mediated by the overproduction of a number of tissue-digesting matrix metalloproteinases (MMPs) such as MMP-2 (type IV collagenase or gelatinase A) and their activators such as membrane-type 1 metalloproteinase (MT1-MMP) [27,28]. Inhibition of MMP-2 in human glioma cell line U-87 results in a dramatic reduction in cell invasion [29], and induction of PKC activation in a D54 human glioblastoma cell line results in enhanced invasion through the activation of several metalloproteases including MMP-2 [30].

Berberine, a natural alkaloid, has been extensively studied and shown to exhibit multiple pharmacological activities such as anti-bacterial [31], anti-oxidative [32], and anti-cancer and anti-inflammation capabilities [32-34]. Berberine also sensitizes human glioma cells to ionizing radiation *in vitro *[35]. Recently, berberine has been shown to exert anti-metastatic properties in non-small lung cancer cells [36], to be cytotoxic to human tumor U937 and murine melanoma B16 cells [37], and to inhibit growth and induce G1 cell cycle arrest followed by apoptosis in human epidermoid carcinoma A431 cells [38].

Although treatment with As_2_O_3 _induces clinical remission in patients with APL without severe toxicity, relapse with As_2_O_3_-resistant cells still occurs [39] and, in addition, As_2_O_3 _remains a toxic compound at relatively high concentrations or exposures [15]. Thus, alternative strategies that would enhance cellular sensitivity to As_2_O_3_, thereby lowering its concentration of action, would be helpful. In the present study, we report for the first time that berberine enhances the antiproliferation activity of As_2_O_3 _in rat glioma and human malignant glioma cell lines. In addition, we investigated As_2_O_3_-mediated suppression of glioma cell invasion which likely involves PKC signaling, ERK phosphorylation, and MMP-2 activation. Thus, this study may have important clinical applications in the design of strategies to treat human glioma.

## Methods

### Cell culture

The rat C6 glioma cell line was originally derived from a N-nitrosomethylurea-induced rat brain tumor [40]. C6 rat cancer cells and U-87 human malignant glioma cells were cultured in minimal essential medium and RPMI (Gibco, Carlsbad, California) supplemented with 10% fetal calf serum (Gibco) and antibiotics (100 units/ml penicillin and 100 μg/ml streptomycin) at 37°C in a humidified atmosphere composed of 5% CO_2 _and 95% air. All experiments were performed in plastic tissue culture flasks, dishes, or in microplates (Nunc, Naperville, Denmark).

### Chemical reagents and antibodies

As_2_O_3 _was obtained from TTY Biopharm (Taipei, Taiwan). Berberine, gelatin, and 3-(4,5-dimethylthiazol-2-yl)-2,5-diphenyl tetrazolium bromide (MTT) were purchased from Sigma-Aldrich (St. Louis, Missouri). Anti-phospho-p44/42 MAPK and horseradish peroxidase-linked anti-rabbit or mouse IgG were from Cell Signaling Technology (Beverly, Massachusetts). Anti-MT1-MMP1, MMP-2, and PKCs α and ε were purchased from Santa Cruz Biotechnology (Santa Cruz, California). Monoclonal anti-ornithine decarboxylase (ODC) antibody was purchased from Sigma-Aldrich.

### Cell growth and proliferation assay

Cell viability was determined using the MTT quantitative colorimetric assay [41]. The cells were seeded at 5 × 10^4 ^cells/ml density and incubated with berberine or As_2_O_3 _at various concentrations (0, 1, 2, 5, 10, and 20 μM) for 24 h. Thereafter, the medium was changed and cells were incubated with MTT (0.5 mg/ml) for 4 h. The viable cell number is directly proportional to the production of formazan, which can be measured spectrophotometrically (λ = 563 nm) upon solubilization with isopropanol. Cell growth was determined by counting the number of cells at indicated periods of time using a Coulter counter and measured using the trypan blue (0.2%) exclusion assay [42].

### Boyden chamber assay

The Boyden chamber assay used for the analysis of tumor cell migration is based on a chamber with two medium-filled compartments. C6 and U-87 glioma cells were allowed to grow as discrete colonies and treated with As_2_O_3 _or berberine as described above. Cells were collected by trypsinization and suspended in serum-free medium at 1 × 10^5^/ml. Migration assays were carried out in a 48-well chemotaxis chamber (Neuro-Probe, Gaithersburg, Maryland). The medium containing 10% fetal calf serum was added to the lower chamber. The lower and upper chambers were separated by an 8 μm pore size polycarbonate membrane (Poretics, Livermore, California). Cells were allowed to migrate for 24 h at 37°C in a humidified atmosphere containing 5% CO_2_. The membrane was fixed in methanol for 10 min and stained with modified Giemsa stain (Sigma-Aldrich) for 1 h. Cells on the upper side of the membrane were removed by cotton swabs. Cells on the lower side of the membrane were counted using a light microscope at 200× magnification. The number of cells that migrated to the lower side of the membrane was determined [36].

### Matrigel invasion assay

Rat C6 and human U87 glioma cells were incubated with Dulbecco's modified Eagle's medium (DMEM) in 10% fetal calf serum and then collected by trypsinization. Cells (1 × 10^5^/ml) in serum-free medium were added to an inner cup of the 48-well Transwell chamber (Corning Life Sciences, Corning, New York) that had been coated with 50 μl of Matrigel (BD Biosciences, Franklin Lakes, New Jersey; 1:10 dilution in serum-free medium). Medium supplemented with 10% serum or indicated agent was added to the outer cup. After 24 h, cells that had migrated through the Matrigel and the 8 μm pore size membrane were fixed, stained, and counted under a light microscope. Each experiment was performed in triplicate.

### Scratch assays

Scratch assays were performed by plating cells in 6-well culture dish. After C6 glioma cells were allowed to attach and reach confluence, a scratch (4 mm) was made through the culture dish. The cells were washed twice with phosphate-buffered saline (PBS, pH = 7) before their subsequent incubation with culture medium in the absence (control) or presence of As_2_O_3 _or combinations at appropriate concentrations. Photographs of treated cells moving within the scratch were taken at the indicated time points. Openlab v3.0.2 image analysis software (Improvision, Coventry, United Kingdom) was used to quantify the area progressively filled with cells over the period of the experiment.

### Preparation of total cell extracts and immunoblot analysis

Cellular lysates were prepared by suspending 1 × 10^6 ^cells in 200 μl of lysis buffer (137 mM NaCl, 15 mM EGTA, 0.1 mM sodium orthovanadate, 15 mM MgCl_2_, 0.1% Triton X-100, 25 mM MOPS, 100 μM phenylmethylsulfonyl fluoride and 20 μM leupeptin, adjusted to pH 7.2). The cells were disrupted by sonication and extracted at 4°C for 30 min. The supernatant was quantitated using the Pierce BCA protein quantitation assay (Pierce, Rockford, Illinois) and were electrotransferred to Immobilon-P membranes (Millipore, Bedford, Massachusetts). Detection of specific proteins was carried out with an enhanced chemoluminescence Western blot kit [43].

### Immunofluorescent staining assay

One day after plating on coverslips, the cells were treated with empty vehicle or indicated agent for appropriate times. Following the treatment period, the cells were fixed in 2% formaldehyde in PBS for 10 min. The cells were then permeabelized in PBS containing 1% bovine serum albumin and 0.1% Triton X-100 for 10 min. The cells were then incubated for 1 h with rabbit anti-F-actin (Abcam, Cambridge, Massachusetts), washed in PBS, and incubated for 1 h with anti-rabbit tetramethylrhodamine isothiocyanate (TRITC) labeled secondary antibodies (Molecular Probes, Carlsbad, California). Following three additional washes, the coverslips were mounted on glass slides in anti-fade medium. The images were then collected using fluorescence microscopy (Carl Zeiss, Oberkochen, Germany).

### PKC translocation assay

Cells were washed twice with PBS and lysed by suspension in 0.5 ml (2 × 10^6 ^cells) of ice-cold homogenization buffer (20 mM Tris· HCl [pH 7.5], 2 mM EDTA, 2 mM EGTA, 6 mM DTT, 50 μg/ml aprotinin, 48 μg/ml leupeptin, 5 μmol/l pepstatin A, 1 mM phenylmethylsulfonyl fluoride). The homogenate was sonicated and then centrifuged for 10 min at 500 × *g*. The supernatant was centrifuged at 100,000 × *g *for 1 h at 4°C. The resulting supernatant was designated as the soluble (cytosol) fraction. The pellet was solubilized in 200 μl of homogenization buffer containing 1% Triton X-100 and centrifuged at 15,000 × *g *for 15 min at 4°C. The supernatant was designated as the particulate (membrane) fraction. All extraction procedures were performed at 4°C. The protein concentration in cell fractions was determined using a commercial protein assay (Bio-Rad, Hercules, California) with bovine serum albumin (BSA) as a standard. Extractions for immunoblotting were based on previously described methods.

### Zymography

MMP-2 and MMP-9 enzymatic activities were assayed by gelatin zymography with some modifications. Culture supernatants were diluted 4:1 with sample 4× buffer (17.4% sodium dodecyl sulphate, 7% sucrose, and phenol red in Tris-HCl, pH 6.8). Sixteen microliters of sample was added to the wells under non-reducing and non-denaturing conditions. After electrophoresis, the gel was washed twice with washing buffer, followed by a brief rinsing in washing buffer with Triton X-100. The gel was then placed in PBS, pH 7.4 containing 0.9 mmol CaCl_2 _and MgCl_2 _and incubated overnight at room temperature on a moving platform. The next morning, the gel was stained with Coomassie Brilliant Blue G250 (0.1% in 25% methanol and 10% acetic acid in water) and destained in the same solution in absence of the dye.

### Statistical analyses

**Figure 1:**
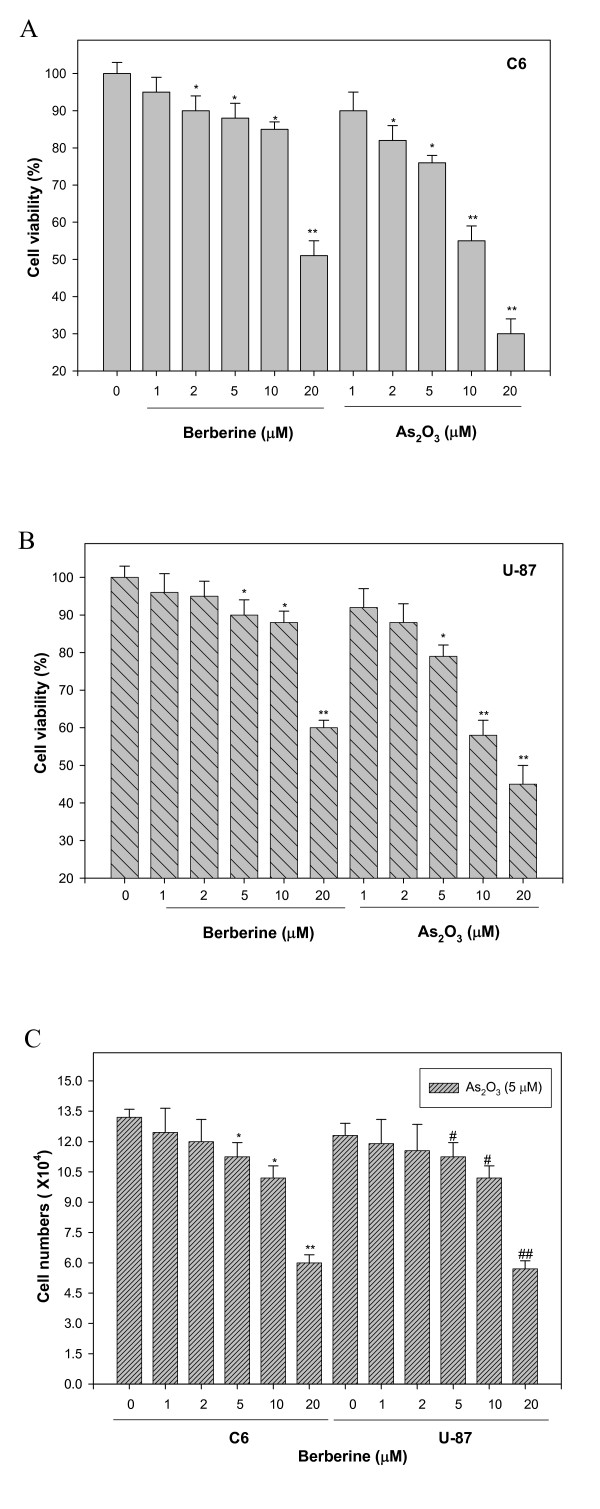
**Evaluation of the cell viability of C6 glioma and human U-87 cells treated with berberine or As_2_O_3_, and co-treated with As_2_O_3 _and berberine for 24 h.** C6 glioma cells (A) and human U-87 cells (B) were incubated with 1, 2, 5, 10, and 20 μM berberine or As_2_O_3 _for 24 h and the proportion of surviving cells was determined using the MTT assay as described in Methods. (C) C6 glioma and human U-87 cells were co-treated with 5 μM As_2_O_3 _and 1, 2, 5, 10, and 20 μM berberine for 24 h, and the cell growth was determined as described in Methods. Control cells were treated with As_2_O_3 _alone. The experiments were performed in triplicate and data are presented as means ± SD. * and ** indicate means that are significantly different when compared to the control group of C6 with *P *< 0.05 and *P *< 0.01, respectively. ^# ^and ^## ^indicate means that are significantly different when compared to the control group of U-87 with *P *< 0.05 and *P *< 0.01, respectively.

**Figure 2:**
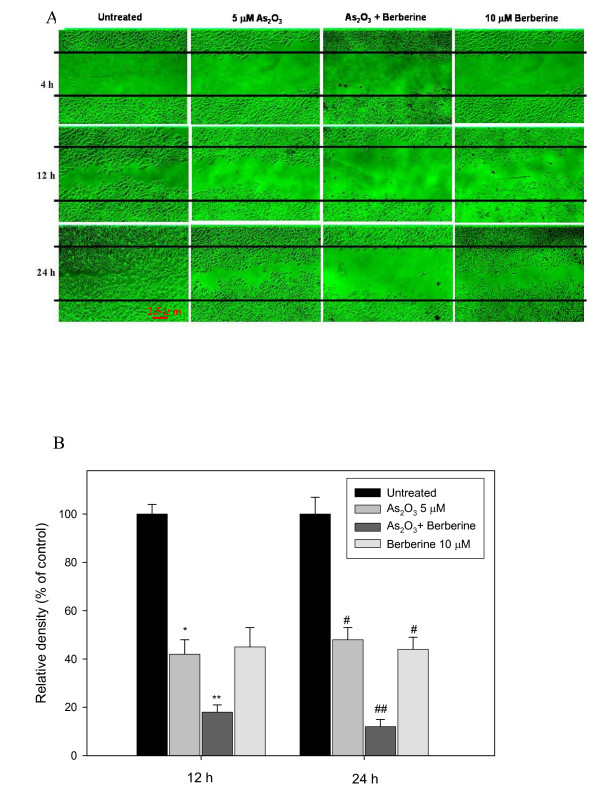
**Determination of the effects of berberine-As_2_O_3 _co-treatment on cell migration and growth using the scratch-wound assay.** C6 glioma cells were incubated with 5 μM As_2_O_3 _or 10 μM berberine alone and with 5 μM As_2_O_3 _and 10 μM berberine for 4, 12, and 24 h and the migration was visualized as described in Methods (A). The percentage of surface area filled by the C6 cells was subsequently quantified by densitometric analyses relative to that of the control which was set at 100% as shown in the graph (B). Data are presented as means ± SD based on three independent experiments. * and ** indicate means that are significantly different when compared to the control group of 12 h incubation with *P *< 0.05 and *P *< 0.01, respectively. ^# ^and ^## ^indicate means that are significantly different when compared to the control group of 24 h incubation with *P *< 0.05 and *P *< 0.01, respectively.

**Figure 3:**
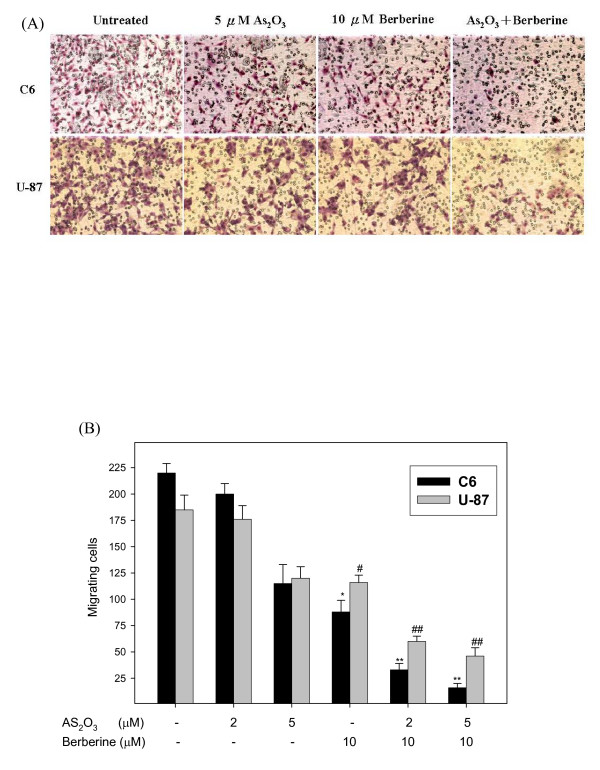
**Effect of berberine on As_2_O_3_-inhibited motility of C6 glioma and U-87 cells.** (A) Cells were incubated with 5 μM As_2_O_3 _or 10 μM berberine and co-treated with 5 μM and 10 μM berberine for 24 h. The lower and upper chemotaxis cells were separated by a polycarbonate membrane. Microscopy images detected cells that migrated into the inner membrane. Magnification: ×200. The cell migration was quantified by counting the number of cells that migrated into the inner membrane (B). Control cells remained untreated. The experiments were performed in triplicate and data are presented as means ± SD. * and ** indicate means that are significantly different when compared to the control group of C6 with *P *< 0.05 and *P *< 0.01, respectively. ^# ^and ^## ^indicate means that are significantly different when compared to the control group of U-87 with *P *< 0.05 and *P *< 0.01, respectively.

**Figure 4:**
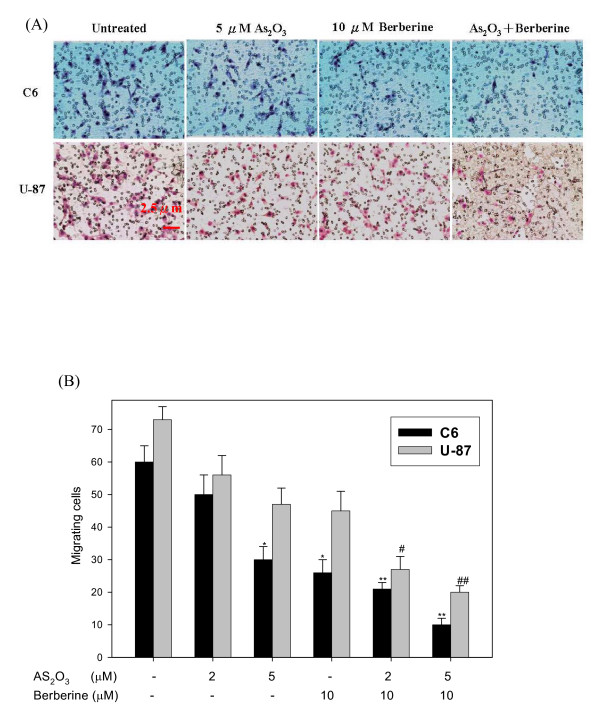
**Effect of berberine on As_2_O_3_-inhibited invasiveness of C6 glioma and U-87 cells.** (A) Cells were incubated with 5μM As_2_O_3 _or 10 μM berberine and co-treated with 5 μM and 10 μM berberine for 24 h. Invasion through a layer of Matrigel was determined by a Boyden Chamber method as described in Methods. The invasiveness was quantified and is presented in the graph (B). Control cells remained untreated. The experiments were performed in triplicate and data are presented as means ± SD. * and ** indicate means that are significantly different when compared to the control group of C6 with *P *< 0.05 and *P *< 0.01, respectively. ^# ^and ^## ^indicate means that are significantly different when compared to the control group of U-87 with *P *< 0.05 and *P *< 0.01, respectively.

**Figure 5:**
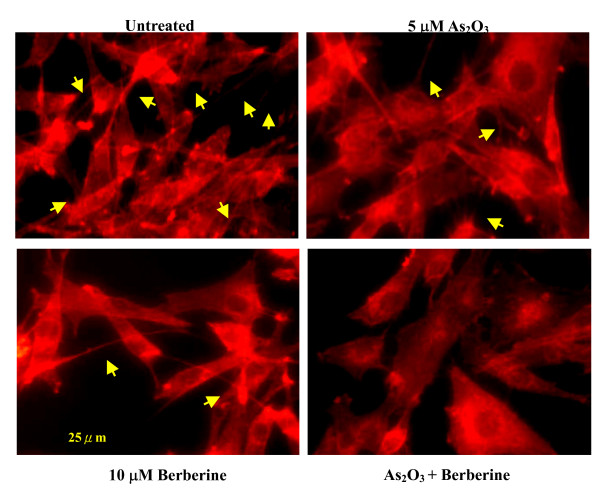
**Actin rearrangements in C6 glioma cells treated with As_2_O_3 _or berberine.** C6 glioma cells were incubated with 5 μM As_2_O_3 _or 10 μM berberine and co-treated with 5 μM and 10 μM berberine for 24 h. Actin rearrangements were visualized by immunolocalization using anti-F-actin antibodies as described in Methods. Co-treatment with berberine and As_2_O_3 _resulted in actin (*arrows*) impolarization at the edges of the cell. Actin ruffing at the edges of the elongated cells was also observed and indicative of increased migration.

**Figure 6:**
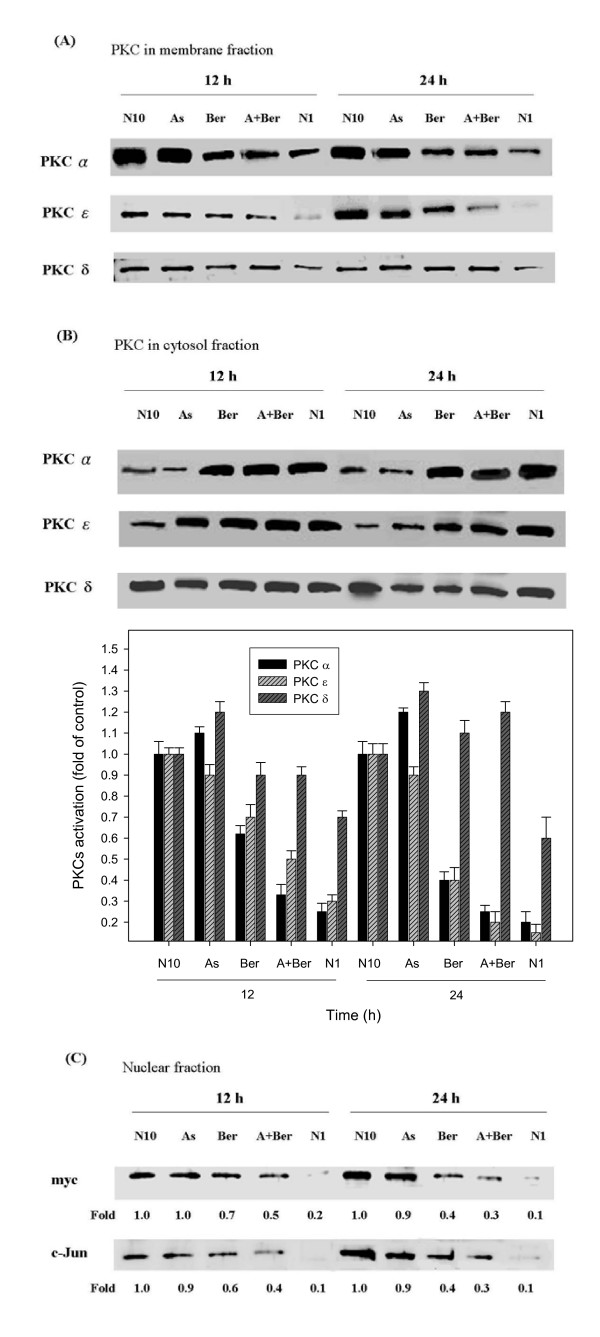
**Effect of berberine and As_2_O_3 _on the translocation of PKCα and PKCε.** C6 glioma cells were incubated for 12 and 24 h with 10 μM berberine (Ber) or 5 μM As_2_O_3 _(As), and with 10 μM berberine and 5 μM As_2_O_3 _(A+Ber) (A, B). Cytosolic (B) and membrane fractions (A) were evaluated for the presence of PKCα and PKCε by Western blotting. Control cells were not treated in the presence of 10% Fetal Calf Serum (FCS) (N10). Cells that were not exposed to agents were used as negative control in the presence of 1% FCS (N1). The PKC levels in the respective fractions were quantified and normalized taking the β-actin value as a loading control as presented in the graph. Each value is the relative ratio of PKCs membrane to cytosol fraction (presumably, the ratio of untreated control is 1). In addition, the nuclear protein fractions were evaluated for the presence of myc and c-jun, two transcription factors that act downstream of PKC, by Western blotting (C). The quantitative data were presented as three repeats from one independent experiment and indications are in panel A. The intensity of the protein bands was quantified and the level relative to the N10 control band was presented (fold).

**Figure 7:**
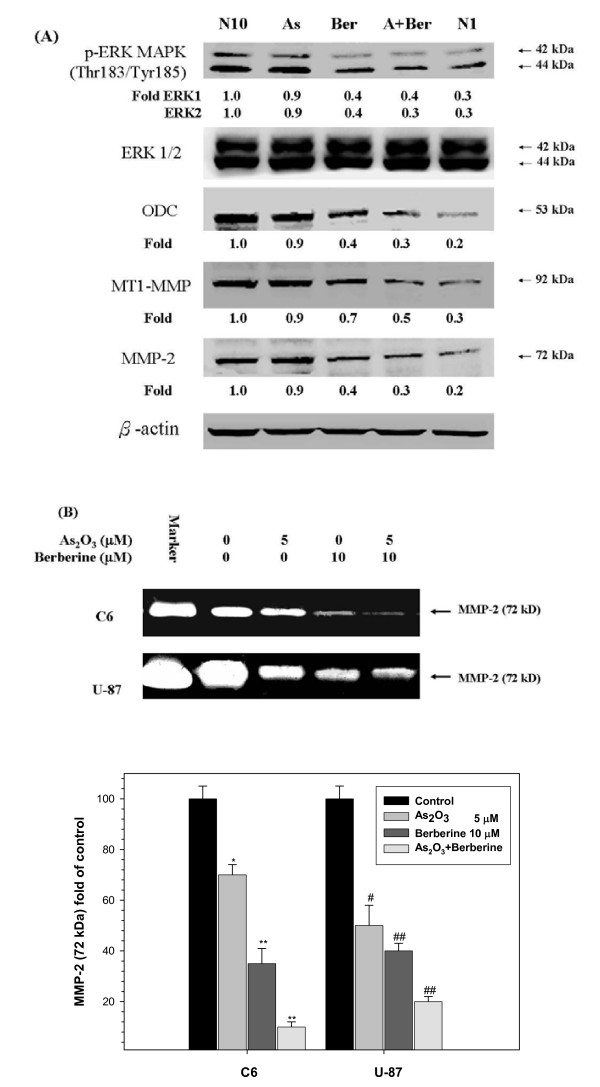
**Berberine and As_2_O_3 _inhibit the phosphorylation of ERK and decrease the ornithine decarboxylase (ODC), MT1-MMP, and MMP2 protein levels.** C6 glioma cells were incubated for 24 h with 10 μM berberine (Ber) or 5 μM As_2_O_3 _(As), and with 10 μM berberine and 5 μM As_2_O_3 _(A+Ber). Total cell lysates were prepared and subjected to Western blot analysis. Protein levels of phosphorylated ERK1/ERK2 and non-phosphorylated ERK1/ERK2, and ODC, MT1-MMP and MMP2 were detected using the respective monoclonal antibodies (A). The condition serum free media were collected for gelatin zymography analysis from C6 glioma and U-87 cells. Determined activities of these proteins were subsequently quantified by densitometric analysis with the value of controls set at 100% as shown in the graph. The quantitative data were presented as the mean of three repeats from one independent experiment. Other data in this figure is presented as mean ± SD of three independent experiments. * and ** indicate means that are significantly different when compared to the control group of C6 with *P *< 0.05 and *P *< 0.01, respectively. ^# ^and ^## ^indicate means that are significantly different when compared to the control group of U-87 with *P *< 0.05 and *P *< 0.01, respectively.

Data are described as the mean ± standard deviation. For Figure 1, each treatment was compared to its relative control using a Student *t *test. For Figures 2, 3, 4, and 5, treatment effects were compared using ANOVA with Bonferroni correction. The latter analysis does not apply to Figure 6, but was used to analyze the data presented in Figure 7. The data were analyzed using the SAS statistical software package "SigmaPlot", version 9.0 (SAS Institute Inc., Cary, North Carolina). The significance level was established at P < 0.05. The quantitative data were presented as three repeats from one independent experiment. This is representative of two independent experiments with similar results.

## Results

### As_2_O_3 _and berberine inhibit the proliferation of C6 and U-87 glioma cells

In the first instance, the effect of As_2_O_3 _and berberine, applied separately or in combination, on the viability of C6 rat glioma cells and U-87 human malignant glioma cells was investigated. The cell proliferation was determined using the MTT assay performed with logarithmically growing glioma cells treated with As_2_O_3 _and berberine (Figures 1A and 1B). Within 24 h of the addition of 5 μM As_2_O_3 _and 10 μM berberine alone on the C6 cells, proliferation was reduced to 78% and 88 %, respectively (*P *< 0.05). Treatment with the same concentrations of As_2_O_3 _or berberine did not elicit marked cytotoxic effects on U-87 cells (Figure 1B). However, at a concentration of 20 μM, decreased cell viability was observed. Interestingly, based on the trypan blue dye exclusion assay, it was observed that 5 μM As_2_O_3 _and 10 μM berberine combined treatment exhibited a minor effect on the viability of C6 and U-87 cells (data not shown). A dose-dependent effect on the inhibition of the C6 or U-87 glioma cell proliferation at lower agent concentrations was observed (Figure 1C). Co-treatment with 5 μM As_2_O_3 _and 10 μM berberine, concentrations used for all further analyses, led to an inhibition of the cell proliferation and indicated no major signs of apoptosis, suggesting that another biological or pharmacological function played a role.

### Berberine enhances As_2_O_3_-mediated reduction in glioma cell migration and invasion

Using the scratch-wound assay, a continuous rapid movement was observed for all cells, but a resultant movement of a glioma cell migration front was clearly evident at 24 h, where a highly confluent (90%–100%) monolayer region gradually migrated into the cell-free 'scratch' region (Figure 2A). In the presence of 5 μM As_2_O_3_, migration was significantly reduced after 12 and 24 h of incubation, whereas co-treatment with 5 μM As_2_O_3 _and 10 μM berberine led to a virtually complete inhibition of cell migration (Figures 2A and 2B).

Cell proliferation and invasive behaviors are important characteristics of cancer cells and indicators of malignance, and both are targets of anti-cancer agent development [44]. The Boyden chamber assay was used to evaluate the inhibitory effect of combined treatment with As_2_O_3 _and berberine on C6 or U-87 glioma cell migration and invasiveness. The observations revealed that berberine-As_2_O_3 _co-treatment resulted in a remarkable inhibition of glioma cell migration as compared to either treatment alone (Figure 3), which supported the results obtained with the scratch-wound assay (Figure 2). In addition, co-treatment with As_2_O_3 _and berberine also exhibited a significant anti-invasive effect on glioma cells (Figure 4), which enhanced the dose-response relationship.

An important characteristic of advanced cancer development metastasis, during which cancer cells migrate to other tissues and organs, is the rearrangement of the cytoskeleton of migrating cells [45]. The cytoskeleton changes in C6 glioma cells treated with 5 μM As_2_O_3 _or 10 μM berberine, and co-treated with As_2_O_3 _and berberine, were visualized using F-actin specific antibodies that bind to F-actin (filopodia; yellow arrow head, Figure 5), and revealed structural rearrangements in the actin cytoskeleton that were observed upon cell spreading and ruffling (Figure 5).

### Berberine prevents cytosol-to-membrane translocation of PKCα and PKCε isoenzymes

Recent studies report that overexpressed or hyperactive PKC is among the most distinguishing characteristics of central nervous system tumors [46]. The PKCα and PKCε activity levels seem to be increased in malignant gliomas and mechanisms related to tumor cell invasion, and metastasis is activated [47,48]. To further evaluate the effect of chemotherapeutic agents such as As_2_O_3 _in the PKC signaling pathway, we examined whether berberine enhanced As_2_O_3_-mediated modulation of the translocation of PKCα and PKCε isoenzymes using Western blot analyses. Remarkably, 10 μM berberine had a dramatic effect on the cytosol-to-membrane translocation of PKCα and PKCε after 12 and 24 h incubation (Figures 6A and 6B). Furthermore, the combined treatment with As_2_O_3 _and berberine resulted in inhibition of the activation of PKCα and PKCε, but not PKCδ (Figures 6A and 6B), as compared to the negative control (1% fetal calf serum). Once activated, PKC can transmit signals to the nucleus via MAPK-mediated cascades and activated ERKs can induce the production of transcription factors including myc and jun [49,50]. To further investigate the effect of As_2_O_3_-berberine co-treatment on PKC-mediated signaling, the nuclear levels of those transcription factors were determined. Figure 6C illustrates that the levels of myc and jun in nuclei were significantly reduced after treatment with 10 μM berberine in the presence of 5 μM As_2_O_3 _for 24 h.

### Inhibitory effect of berberine and A_2_O_3 _on the activation of MMP-2 associated with invasiveness of glioma cells via interference with PKCs

Activated PKCs enable the expression of genes encoding enzymes that are involved in cell proliferation and invasion [30,46,50] such as the gene encoding ornithine decarboxylase (ODC), an enzyme that plays a role in cell transformation and excessive extracellular matrix degradation [51]. Because the observations presented above strongly suggest that berberine and As_2_O_3 _act via the inhibition of PKC signaling, the activation of three PKC-dependent key factors was examined by Western blot analyses. These experiments demonstrated that the berberine-As_2_O_3_-mediated enhanced suppression of ERK phosphorylation led to a decrease of the levels of ODC, MT1-MMP, and MMP-2 in C6 glioma cells to values that resembled those observed in negative controls (Figure 7A).

Extracellular matrix breakdown is pivotal for cellular invasion, indicating that matrix-degrading proteinases are essential for tumor cell metastasis [28]. Therefore, we determined the activity of MMP-2 by gelatin zymography after 24 h incubation with 5 μM As_2_O_3 _and 10 μM berberine in C6 or U-87 glioma cells. A significant decrease in the activity of MMP-2 was observed after 24 h exposure to As_2_O_3 _and berberine (Figure 7B), likely explaining why co-treatment with berberine and As_2_O_3 _strongly affects glioma cell migration and invasiveness.

## Discussion

Berberine is a naturally occurring alkaloid that exhibits various pharmacological effects. Clinical trials and studies in animal model systems have demonstrated that berberine has anti-microbial, vulnerary, cardiovascular [52], immunostimulatory, anti-hemorrhagic, and anti-inflammatory properties, as well as selective repression of the growth of several carcinoma cells without causing cytotoxicity in the liver [32,53]. Control experiments using rat primary cortical astrocytes illustrated that treatment with 5 μM As_2_O_3 _or 20 μM berberine, or co-treatment with 5 μM As_2_O_3 _and 20 μM berberine, did not cause any detectable toxic effects (data not shown). However, the effect of berberine on migration and invasion of glioma cells at relatively low concentrations was striking, particularly in the presence of As_2_O_3_. Most interesting was the enhanced effect that As_2_O_3_-berberine co-treatment had on glioma cell migration and invasiveness, as 10 μM berberine significantly lowered the concentration of As_2_O_3 _required to obtain an antiproliferation effect on human glioma cells. This dose-lowering effect, unique to this study, could not be exploited in other studies in which tumor cells were treated with either berberine [37,38] or As_2_O_3 _[14].

As_2_O_3 _has considerable efficacy in the treatment of relapsed APL, inducing partial differentiation and promoting apoptosis of malignant promyelocytes [54,55]. The mechanisms underlying As_2_O_3_-mediated apoptosis are only beginning to be understood, but appear to be distinct from those employed by traditional cytotoxic agents. The intracellular glutathione redox system represents the best-characterized mechanism of As_2_O_3 _sensitivity [39,55]. These findings underscore the importance of understanding how differences in cell types or cellular environments might affect the action of As_2_O_3_. A determination of the factors that mediate sensitivity to As_2_O_3 _will allow the use of this agent in such a way as to optimize therapeutic outcomes and minimize toxicity in the treatment of various malignancies. To date, evidence that the development of resistance to chemotherapy affects the response to arsenic is scarce. Thus, As_2_O_3 _represents a novel chemotherapeutic agent worth for continuous investigation, particularly when used in combination with another agent such as berberine that exhibits an enhanced inhibitory effect, which allows lowering the working As_2_O_3 _concentration.

In previous studies, cell movement through tissue has been observed to play a primary role in cancer progression. This process requires a series of distinct, but concerted, biological events in which the actin cytoskeleton plays an essential role. These events include tumor cell attachment to extracellular matrix (ECM) components, and the degradation of the matrix by tumor cell-associated proteases [28,56]. It has long been known that in most cell types, one or more PKC isoforms influence the morphology of the F-actin cytoskeleton and overexpressed or hyperactive PKC is among the most distinguished characteristics of malignant central nervous system tumors including C6 or U-87 glioma cells [46,57]. In certain tumors, a reduction in PKCα and PKCε expression merely results in growth inhibition, so that a cytotoxic effect requires induction of apoptosis through activation of PKCδ [58]. Exposure of C6 cells in the presence of As_2_O_3 _to berberine exhibits a significant effect characterized by alterations in cell shape and actin cytoskeleton changes. Moreover, the data presented herein suggests that the cytosol-to-membrane translocation of PKCα and PKCε is slightly suppressed by As_2_O_3_, and this inhibition is enhanced upon co-treatment with berberine for 24 h, although berberine itself did cause inactivation of PKCα and PKCε but not PKCδ. Similar to the action of the PKC signaling pathway, the expression on myc, c-fos, ODC, MT1-MMP, and MMP-2 as well as the phosphorylation of ERK by co-treatment could be abolished.

Arsenic-containing compounds have been used for the treatment of cancer for hundreds of years in both traditional Chinese and Western medicine [59]. Only a few reports are available that describe the underlying mechanisms of the action of specific chemotherapeutic agents used to treat cancer metastasis. In this study, we demonstrate for the first time that berberine sensitizes malignant glioma cells for As_2_O_3_-mediated suppression of migration and invasiveness likely via decreased PKC signaling and MMP-2 activation in the extracellular matrix. The involvement of PKC signaling is not yet undoubtedly proven, as the effect of As_2_O_3 _and berberine on glioma tumor cells overexpressing PKCα and PKCε remains to be investigated. Furthermore, we tried to investigate the dual effect that berberine has on glioma cells. First, berberine exhibits an anti-oxidant activity thereby protecting healthy cells against the As_2_O_3_-induced production of toxic reactive oxygen species [5,33,53]. Second, berberine may reduce the As_2_O_3_-mediated carcinogenic effect by inhibiting PKCα translocation in the mouse epidermal JB6 cells [60]. This dual effect is particularly important in a co-treatment with As_2_O_3 _because As_2_O_3 _is a carcinogen [10]; however, upon co-treatment with berberine, the working concentration of As_2_O_3 _can be significantly lowered, while, in addition, healthy cells are protected against possible adverse side effects caused by exposure to As_2_O_3_.

Invasion and metastasis inhibitors are effective (alone or in combination therapy with other agents) in restraining new tumor formation when earlier therapy or surgery has failed. Therefore, it appears that a combination of As_2_O_3 _and berberine inhibits the proliferation and invasion of glioma cells. Although future studies are required to experimentally link the various pathways that appear to be involved in the berberine/As_2_O_3_-mediated suppression of glioma cell migration and invasiveness, to better understand the molecular mechanisms that occur in the extracellular matrix surrounding glioma cells during tumor metastasis, to investigate novel methods to specifically deliver, for instance, berberine and As_2_O_3 _to glioma tumor cells, and to investigate the effectiveness and safeness of berberin-As_2_O_3 _co-treatment in a clinical setting, this study forms the basis for the design of novel therapeutic strategies to ameliorate and prevent human glioma formation. Furthermore, the observations presented herein suggest that (i) berberine should be further considered as a complementary antitumor treatment in clinical applications, and (ii) application of berberine has a dual ameliorating effect as it enhances the antitumor effect of As_2_O_3 _while it allows a lower working concentration of As_2_O_3 _by which its global carcinogenic effect to healthy cells, mediated by the fact that elemental arsenic can enter the cerebrospinal fluid [61], is diminished.

## Conclusion

We demonstrate that berberine enhances inhibition of the migration and invasiveness of glioma cells mediated by As_2_O_3_, or, in other words, that a concentration as low as 10 μM of the natural compound berberine is sufficient to significantly lower the As_2_O_3 _concentration needed to obtain its antiproliferation effect in a rat and human malignant glioma cell line. While not yet undoubtedly proven, our results suggest that this effect is likely mediated by the disturbance in PKC signaling which leads to a reduction of the level of MMP-2, a metalloprotease involved in cancer cell migration during metastasis.

## List of abbreviations

APL, acute promyelocytic leukemia; ECM, extracellular matrix; ERK, extracellular signal-regulated kinase; MAPK, mitogen-activated protein kinase; MMP, matrix metalloproteinase; MT1-MMP, membrane type-1 matrix metalloproteinase; ODC, ornithine decarboxylase; PBS, phosphate-buffered saline; PKC, protein kinase C

## Competing interests

The author(s) declare that they have no competing interests.

## Authors' contributions

LTH conceived, designed, and performed the study, and prepared the manuscript. KHC performed data analysis, and provided cell lines and technical assistance to LTH. Chou FP supervised work and manuscript review. LFJ, supervised the entire study and manuscript revision.

## Pre-publication history

The pre-publication history for this paper can be accessed here:


